# Synthesis and Biological Evaluation of Novel Cationic Rhenium and Technetium-99m Complexes Bearing Quinazoline Derivative for Epidermal Growth Factor Receptor Targeting

**DOI:** 10.3390/pharmaceutics16091213

**Published:** 2024-09-16

**Authors:** Sotiria Triantopoulou, Ioanna Roupa, Antonio Shegani, Nektarios N. Pirmettis, Georgia I. Terzoudi, Aristeidis Chiotellis, Maria Tolia, John Damilakis, Ioannis Pirmettis, Maria Paravatou-Petsota

**Affiliations:** 1 Department of Medical Physics, School of Medicine, University of Crete, P.O. Box 2208, 71003 Heraklion, Greece; iro@rrp.demokritos.gr (S.T.); john.damilakis@med.uoc.gr (J.D.); 2 Institute of Nuclear and Radiological Sciences and Technology, Energy & Safety, NCSR “Demokritos”, P.O. Box 60037, 15310 Athens, Greece; ioanna_roupa@yahoo.gr (I.R.); ant.she@hotmail.gr (A.S.); nnpirmettis@pharm.uoa.gr (N.N.P.); gterzoudi@rrp.demokritos.gr (G.I.T.); achiotel@rrp.demokritos.gr (A.C.); 3 Department of Radiation Oncology, University Hospital of Iraklion, 71110 Iraklion, Greece; mariatolia@uoc.gr

**Keywords:** anticancer activity, epidermal growth factor receptor inhibitors, rhenium, technetium, A431, radiation

## Abstract

**Background/Objectives:** Epidermal growth factor receptor (EGFR) plays a vital role in cell proliferation and survival, with its overexpression linked to various malignancies, including non-small cell lung cancer (NSCLC). Although EGFR tyrosine kinase inhibitors (TKIs) are a key therapeutic strategy, acquired resistance and relapse remain challenges. This study aimed to synthesize and evaluate novel rhenium-based complexes incorporating EGFR TKIs to enhance anticancer efficacy, particularly in radiosensitization. **Methods:** We synthesized a rhenium tricarbonyl complex (Complex **2**) and its ^99m^Tc analog (Complex **2**’) by incorporating triphenylphosphine instead of bromine as the monodentate ligand and ${PF}_{6}^{-}$ as the counter-ion, resulting in a positively charged compound that forms cationic structures. Cytotoxicity and EGFR inhibition were evaluated in A431 cells overexpressing EGFR using MTT assays, Western blotting, and flow cytometry. Radiosensitization was tested through MTT and clonogenic assays. The ^99m^Tc complex’s radiochemical yield, stability, and lipophilicity were also assessed. **Results:** Complex **2** exhibited significant cytotoxicity with an IC_50_ of 2.6 μM and EGFR phosphorylation inhibition with an IC_50_ of 130.6 nM. Both complex **1** and **2** induced G_0_/G_1_ cell cycle arrest, with Complex **2** causing apoptosis. Radiosensitization was observed at doses above 2 Gy. Complex **2**’ demonstrated high stability and favorable lipophilicity (LogD_7.4_ 3.2), showing 12% cellular uptake after 30 min. **Conclusions:** Complexes **2** and **2**’ show promise as dual-function anticancer agents, offering EGFR inhibition, apoptosis induction, and radiosensitization. Their potential as radiopharmaceuticals warrants further in-depth investigation in preclinical models.

## 1. Introduction

Epidermal growth factor receptor (EGFR) is the first member of the ErbB family, consisting of four receptor tyrosine kinases, with an essential role in cell growth, proliferation, survival, differentiation, adhesion, and migration [1,2]. The homo or heterodimerization of the receptor by specific ligands leads to its phosphorylation and activation of downstream signaling pathways related to cell proliferation and survival, such as the RAS-RAF-MEK-ERK and PI3K-AKT-mTOR pathways [3].

Although EGFR expression is a significant component in the physiological function of healthy cells, the receptor is also involved in the pathogenesis of many malignancies [4]. Overexpression, gene amplification, or mutations in EGFR are correlated with poor prognosis and clinical outcome in many types of cancer, such as lung, breast, brain, head and neck, ovarian, cervical, bladder, and esophageal cancer [5,6,7]. For this reason, targeted treatment options against EGFR have been developed and are clinically used currently, especially in cases of non-small cell lung cancer (NSCLC), the most common type of lung cancer [8]. Among the available therapeutic strategies, tyrosine kinase inhibitors targeting EGFR (EGFR TKIs) have been the primary therapeutic option for the last twenty years [2,7]. Today, three generations of EGFR TKIs are clinically approved, with the most widely used being gefitinib, erlotinib, afatinib, and dacomitinib [9]. These inhibitors are quinazoline derivatives that bind to the intracellular domain of EGFR and inhibit its phosphorylation and further activation of the signaling cascades [10]. In the literature, EGFR TKIs are often reported to be synergistic with radiation and act as radiosensitizers [11,12,13,14,15]. 

Although EGFR TKIs are the treatment of choice, especially for NSCLC, relapse and drug resistance are observed in many patients after treatment [16]. This evolved acquired resistance is characterized by various on-target mutations and off-target resistance mechanisms [17]. To solve this problem, along with the occasionally severe side effects observed, the introduction of new pharmacophores acting as dual-target inhibitors has been proposed [18]. In this category, the development of pharmacophores that combine metal complexes with EGFR TKIs may be promising novel multi-target anticancer drugs, resulting in a combination of different mechanisms of action [19]. Several metals have been investigated in combination with EGFR TKIs, such as Ruthenium, Cobalt, Platinum, Rhodium, Iron, and Gallium [19]. Among the different metals, Rhenium complexes seem to be promising, since they are known to act as anticancer agents through several mechanisms such as targeting cancer cell organelles, interacting with DNA, inhibiting enzymes and protein kinases, disrupting mitochondria activity, and inducing cytoplasmic vacuolization and apoptosis [20,21].

**Figure 1:**
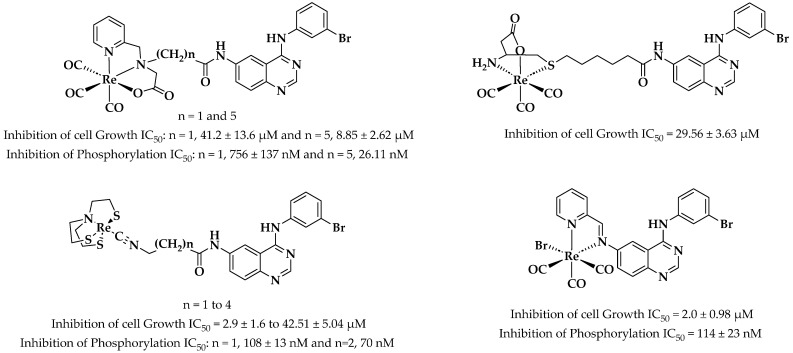
Inhibition of epidermal growth factor receptor (EGFR) phosphorylation and inhibition of A431 cell growth of quinazoline derivatives present in the literature [22,23,24,25].

In previous works, Rhenium and Technetium-99m complexes bearing quinazoline derivatives were synthesized and characterized primarily as biomarkers for the molecular imaging of tumors over-expressing EGFR [22,23,24,25] (Figure 1). These efforts aimed to contribute to addressing the clinical need for the accurate and reliable prediction of the patient sensitivity to anti-EGFR drugs based on the development of radionuclide-based imaging agents [24]. The anilinoquinazoline pharmacophore was attached to tridentate or monodentate ligands via a short spacer to synthesize these complexes. Alternatively, an anilinoquinazoline moiety was incorporated into a bidentate chelator with the sixth coordination site occupied by a bromine atom, resulting in a neutral complex with notable anticancer activity [25]. In this study, the bromine atom was replaced with triphenylphosphine (PPh_3_) to form Complex **2**, yielding a positively charged compound with ${PF}_{6}^{-}$, as the counter-ion. The new complex was evaluated for its cytotoxic effect and EGFR-targeting capability. Additionally, the effect of complexes **1** and **2** on the cell cycle and the radiosensitizing properties in combination with radiation were investigated using the A431 cell line overexpressing EGFR. Furthermore, the synthesis, characterization, and preliminary evaluation of an analogous cationic ^99m^Tc-Complex **2**’ is reported, underscoring its potential for radiopharmaceutical applications.

## 2. Materials and Methods

All reagents and starting materials were purchased from commercial suppliers and used without further purification. Rhenium carbonyl was purchased as Re_2_(CO)_10_ and converted to [NEt_4_]_2_[Re(CO)_3_Br_3_], according to the literature [26]. The 6-(pyridine-2-methylimine)-4-[(3-bromophenyl)amino]-quinazoline and *fac*-[Re(CO)_3_(imQz)Br] (complex **1**) were synthesized according to the literature [25]. IR spectra were recorded on a Brucker™ Alpha II™ FT-IR/ATR Spectrometer (Bruker, Billerica, USA) between 4000 and 400 cm^−1^. NMR spectra were obtained in DMSO-d_6_ at 25 °C on a Bruker Avance DRX 500 MHz (^1^H at 500.13 MHz) (Bruker, Billerica, USA). The measured chemical shifts are reported in *δ* (ppm), and the residual signal of the solvent was used as the internal calibration standard (DMSO-d_6_: ^1^H = 2.50 ppm). HPLC analysis was performed on Waters 600 system equipped with a Waters 2487 Dual λ absorbance detector and a Gabi γ-detector. Separations were accomplished using a Macherey-Nagel Nucleosil C-18 RP column of 150×4 mm, 5 μm, eluting with a binary gradient system including water (0.1% trifluoroacetic acid (TFA)) (solvent A) and methanol (0.1% TFA) (solvent B) as follows: 0–1 min, 5% B; 1–8 min, 5–85% B; 8–20 min, 85% B; 20–30 min, 95% B. Flow rate, 1 mL/min; UV detection, 254 nm.

### 2.1. Synthesis of Re-Complex ***2***

*fac-[Re(CO)_3_(imQz)(pph_3_)](PF_6_) (**2**)*. To a solution of complex **1** (49.0 mg, 0.065 mmol) in 30 mL acetonitrile, AgPF_6_ (18.1 mg, 0.072 mmol) was added, and the solution was refluxed overnight. Then, the forming AgBr was filtered through celite, and PPh_3_ (26.2 mg, 0.10 mmol) was added to the remaining solution. The reacting mixture was refluxed for 3 h. Then, the volume of the solution was reduced to 5 mL, followed by adding 30 mL of diethyl ether. The resulting precipitating orange solid was filtered under vacuum and washed several times with diethyl ether. Yield: 42 mg, 60%. HPLC: t_R_ = 13.3 min. IR (cm^−1^): 2037 (s), 1956 (sh, s) and 1914 (br, s) (*fac*-Re(CO)_3_), 691 and 743 (PPh_3_), and 556 and 833 (${PF}_{6}^{-}$). ^1^H NMR (500 MHz, DMSO-d_6_) δ_H_ 9.7 (1H, H-6), 9.4 (1H, H-1), 8.8 (1H, H-11), 8.6 (1H, H-14), 8.3 (1H, H-4), 8.2 (1H, H-3), 8.1 (1H, H-20), 8.0 (1H, H-8), 7.9 (1H, H-9), 7.8 (1H, H-2), 7.7 (1H, H-16), 7.5 (1H, H-17), 7.5 (2H, H-18), 7.3 (3H, PPh_3_ *para*), 7.2 (6H, PPh_3_ *meta*), and 7.1 (6H, PPh_3_ *ortho*).

### 2.2. Synthesis of ^99m^Tc-Complex ***2***’

Caution! All manipulations involving ^99m^Tc radioactive solutions were conducted by authorized personnel in a licensed laboratory equipped with adequate lead shielding to ensure safety.

Sodium pertechnetate ([^99m^Tc]NaTcO_4_) was obtained from a ^99^Mo/^99m^Tc generator eluate (Ultra-Technekow™ V4 Generator, Curium Pharma, Petten, Netherlands) in physiological saline with an activity range of 370 to 740 MBq/mL. The precursor *fac*-[^99m^Tc(CO)_3_(H_2_O)_3_]^+^ was synthesized according to previously established protocols [23]. 

*fac-^99m^Tc(CO)_3_(imQz)(PPh_3_)]^+^ (**2’**)*. To a sealed glass vial containing a freshly prepared solution of *fac*-[^99m^Tc(CO)_3_(H_2_O)_3_]^+^ (450 μL, 37–370 MBq, pH 6), 450 μL of an methanol solution of imQz (2 × 10^−2^ M) was added. The vial was flushed with nitrogen and incubated at 40 °C for 30 min. Subsequently, 100 μL (10^−2^ M) of the monodentate ligand PPh_3_ in methanol was added to the mixture and allowed to react at room temperature for 30 min. The reaction was monitored using RP-HPLC, which showed the formation of a single complex with a t_R_ of 13.9 min and a RCY of 85%. The characterization of the complex was confirmed using a comparative RP-HPLC analysis using the analogous rhenium complex *fac*-[Re(CO)_3_(imQz)(PPh_3_)]^+^ (**2**) as a reference (HPLC: t_R_ = 13.3 min).

### 2.3. Stability Studies of ^99m^Tc-Complex ***2***’

The stability was evaluated by incubating HPLC-purified samples in PBS (pH 7.4) at room temperature for 6 h. To challenge the stability, 0.5 mL of an aqueous solution of 2 × 10^−3^ M cysteine or histidine was added to 0.5 mL (3.7 MBq) of ^99m^Tc-complex solution and incubated at 37 °C for 6 h. Aliquots of these solutions were analyzed using HPLC at 1, 3, and 6 h [27]. 

### 2.4. Lipophilicity Study of ^99m^Tc-Complex ***2***’

The lipophilicity was determined using the shake flask method. Briefly, 50 μL (~74 kBq) of the isolated complex was added to a centrifuge tube containing 4 mL of a 1-octanol/PBS (0.1 M, pH 7.4) (1:1) mixture. After vortexing for 1 min at room temperature, the mixture was centrifuged at 5000 rpm for 5 min. The radioactivity of three aliquots (3 × 0.1 mL) from the 1-octanol and PBS phases was measured using a gamma counter. The 1-octanol aliquot of 0.5 mL was repartitioned until consistent values were achieved. The measurement was repeated three times. The distribution coefficient (D_o/w_) values were calculated by dividing the counts in the 1-octanol phase by those in the PBS phase [28]. 

### 2.5. In Vitro Biological Studies

Cell line: The in vitro assays were performed using the human epidermoid carcinoma A431 cell line, overexpressing EGFR. This cell line was kindly provided by Dr Mishani (Hebrew University, Hadassah University Hospital Campus, Department of Medical Biophysics and Nuclear Medicine, Jerusalem, Israel). Cells were cultured in Dulbecco’s Modified Eagle Medium with L-glutamine, supplemented with 10% fetal bovine serum and penicillin/streptomycin at final concentrations of 100 UI/100 μg per mL. Cell cultures were maintained at 37 °C in a humidified environment of 5% CO_2_.

#### 2.5.1. Cytotoxicity and Growth Inhibition (MTT Assay)

The cytotoxicity of the new complexes was evaluated using an MTT assay [23]. Cells were seeded in 96-well plates (4000 cells/well) and were incubated for attachment. The day after, the medium was removed and replaced by various concentrations of complex solutions (1 mM, 100 μΜ, 10 μΜ, 1 μΜ, 100 nM, and 10 nM in complete medium). The final concentration of Dimethyl Sulphoxide (DMSO) never exceeded 1%. Controls were considered to be cell cultures without compound treatment. Four replicates were performed for each concentration. After an incubation period of 72 h, the supernatants were removed and replaced by a 100 μL/well of MTT (Sigma, St. Louis, MO, USA) solution (1 mg/mL of medium). Four hours later, the MTT solution was removed and replaced by 2-propanol. After thorough shaking to solubilize the formed MTT crystals, the absorbance of each well was measured in an ELISA reader at the test and reference wavelengths of 540 and 620 nm, respectively. The mean value of the optical density (OD) and the percentage of the OD decreases (mean of the OD of four replicates/mean OD of the control) were calculated for each compound concentration. The OD percentage against compound concentration was plotted in a semilog chart using the PRISM software (PRISM 8, version 8.4.0, 2020), and the cytotoxicity half-maximal inhibitory concentration (IC_50_) value was determined from the created dose–response curve. This assay was performed in triplicate. 

#### 2.5.2. Inhibition of EGFR Phosphorylation

The capability of the new complex to target EGFR and inhibit its phosphorylation was tested using the Western blotting technique, as previously described [24]. Briefly, cells (5 × 10^5^ cells/well) were seeded in 6-well plates and were incubated for attachment. The day after, the medium was replaced by fresh medium without fetal bovine serum and cells were incubated for another 24 h. The following day, the compounds were added to the cultures at various final concentrations ranging from 0.1 nM to 10 μΜ with 1% maximum DMSO concentration. The cells were incubated with the compounds for 2 h and then for 5 min more with EGF (Cell Signaling, hEGF) at a final concentration of 20 ng/mL. Cells without compound treatment, with or without the presence of EGF, were considered to be positive and negative controls, respectively. Later on, cells were washed with PBS, lysed with cell lysis buffer (50 mM Tris-HCl pH 7.4, 150 mM NaCl, 1% Triton X-100, 0.25% *w*/*v* sodium deoxycholate, 0.1% *w*/*v* sodium dodecyl sulfate, protease and phosphatase inhibitors with EDTA) and harvested. An amount of 10 μg total protein from each sample, as estimated using the Bradford method, and 5 μL of molecular weight marker were loaded onto an 8% polyacrylamide gel for electrophoresis. After protein separation using electrophoresis, the protein samples were transferred to a nitrocellulose membrane. The membrane was then blocked for one hour in 5% non-fat dry milk in a TBST-T buffer (Tris-buffered saline and Tween 20). Afterward, the membrane was incubated for two hours in the primary phosphotyrosine antibody (Cell Signaling, Danvers, MA, USA, p-Tyr 100, mouse mAb, 1:1000) and later on for 1 h in the secondary anti-mouse IgG antibody diluted (Cell Signaling, HRP Linked, 1:1000). The detection of EGFR phosphorylation was performed with the use of a chemiluminescent detection system (ECL kit, Amersham, Uppsala, Sweden) according to the manufacturer’s instructions. Protein band quantification and the percentage of EGFR phosphorylation inhibition were calculated using the Image-J software 1.53v. The EGFR phosphorylation inhibition IC_50_ value was determined from the dose–response curve created using the PRISM software. This assay was performed in duplicate.

#### 2.5.3. Cell Cycle Analysis

Cell cycle analysis was performed using flow cytometry. For this purpose, cells were cultured in 6-well plates (5 × 10^5^ cells/well), incubated overnight, and then treated for 24 h with the complexes at the cytotoxicity IC_50_ concentration. Cells were then harvested, and the suspended cell pellet was fixated using 70% ethanol and stored at 4 °C. The day after fixation, cells were centrifuged and resuspended in propidium iodide (PI, Sigma) staining solution (50 μg/mL PI, 10 mM Tris-HCl., 5 mM MgCl_2_, 10 μg/mL RNAse A in Phosphate-Buffered Saline-PBS) and incubated at 4 °C in the dark for 30 min. Flow cytometry was performed using a FACS Calibur (Becton Dickinson, San Jose, CA, USA) flow cytometer using the ModFit v6 software (Verity Software House, Topsham, ME, USA). This experiment was performed in triplicate.

#### 2.5.4. Radiosensitivity: MTT Assay and Clonogenic Assay

MTT assay: To evaluate the combined action of radiation with the complexes on the A431 radioresistant cell line, the MTT method was used [29,30]. Cells were seeded into 96-well plates (1000 cells/well). The day after seeding, the medium was removed, and the complexes were added at 1μΜ concentration. Cultures without treatment were also included as controls. Each experimental point was repeated in tetraplicate. DMSO was present at constant concentration (0.2%) in all experimental points. The cultures were maintained for 24 h. Cells were then irradiated with 0, 1, 2, and 4 Gy using a Co-60 Nordion GammaCamber 4000A irradiator at a dose rate of 1 Gy/min. The medium was refreshed after irradiation. The cultures were maintained for 7 days. Afterward, the supernatant was removed and the cells were exposed to 1 mg/mL MTT solution for 4 h at 37 °C. The MTT solution was removed and replaced by 2-propanol. After thorough shaking, the absorbance of each well was measured in an ELISA reader, as previously described. The experiment was performed in tetraplicate.

Clonogenic assay (Colony formation assay): The drug–ionizing radiation interaction was tested by investigating the ability of a single cell to grow into a colony [31]. A431 cells were seeded in 6-well plates (1000 cells/well) for attachment. The next day, complexes were added at a final concentration of 1 μM. Dimethyl sulphoxide (0.2%) was present at constant concentration in all experimental points. Twenty-four hours after the treatment, cells were irradiated at 0, 2, 4, 6, 8, and 10 Gy using a Co-60 Nordion GammaCamber 4000A irradiator at a 1 Gy/min dose rate. The medium was refreshed every three days while cells were incubated at 37 °C 5% CO_2_ for 14 days. Colonies were then fixed and stained with crystal violet. Colonies (50 cells or more) were counted using the optical microscope. Survival fractions were calculated and plotted as a function of the radiation dose on a semi-log scale. The experiment was performed in triplicate.

#### 2.5.5. Cell Uptake of ^99m^Tc-Complex **2**’

The uptake of complex **2**’ by A431 cells was assessed in vitro as a function of time. A431 cells (10^6^ cells/well) were seeded in 6-well plates and incubated for 24 h. The cells were then treated with complex **2**’ (37 kBq) in a culture medium for 1 h at 37 °C. Experiments were performed in triplicate. Following incubation, cells were washed three times with serum-free medium to remove unbound compound. The cells were then trypsinized with 1 mL of trypsin-EDTA solution (0.05% trypsin, 0.02% EDTA) for 15 min at 37 °C. The cell suspensions were transferred to scintillation vials, cell-associated radioactivity was measured using an automated gamma counter, and the percentage of cell-bound radioactivity was calculated. The experiments were performed in triplicate.

## 3. Results and Discussion

### 3.1. Synthesis of Re-Complexes

**Scheme 1:**
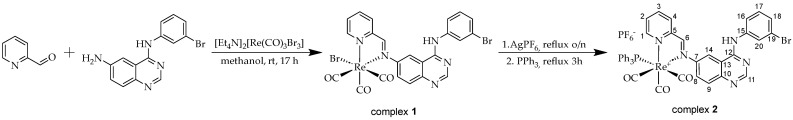
Synthetic procedure of the studied complexes, **1** and **2.** The numbering on the complex **2** structure is used in the NMR assignments.

Rhenium complex **2** was synthesized as presented in Scheme 1. First, the rhenium precursor, [NEt_4_]_2_[Re(CO)_3_ Br_3_], reacts with the pyridine-2-carboxaldehyde, which coordinates with nitrogen and oxygen atoms, and then the 6-amino-4-[(3-bromophenyl)amino] quinazoline [32] as added forming the complex **1**. After treatment with AgPF_6_ in CH_3_CN, followed by the addition of PPh_3_, the bromine axial ligand is removed as AgBr, and the coordination sphere of rhenium is completed by PPh_3_, forming complex **2** with good yields (60%). Complex **2** was characterized using IR and NMR spectroscopy. The purity of the complexes was also confirmed with HPLC. Complex remain stable in solution for months, as shown with HPLC and NMR. The IR spectra of complex **2** exhibited the characteristic peaks of the fac-[Re(CO)_3_]^+^ core, from 2037 to 1914 cm^−1^, due to the C≡O stretching vibrations [33]. The two bands due to the PPh_3_ were observed at 691 cm^−1^ and 743 cm^−1^. Two bands due to the ${PF}_{6}^{-}$ counter ion were observed at 556 cm^−1^ and 833 cm^−1^. The successful coordination of the NN bidentate ligand bearing the pharmacophore and the monodentate ligand was also confirmed using NMR spectroscopy (Supplementary Materials). Upon coordination of PPh_3_, characteristic shifts are noted for protons of the pyridine moiety and the imine proton, as expected.

### 3.2. Radiochemistry and In Vitro Evaluation

The fac-[^99m^Tc(CO)_3_(imQz)(PPh_3_)]^+^ complex **2**’ was synthesized with a high Radiochemical Yield (RCY) of 85%. Reverse Phase High-Pressure Liquid Chromatography (RP-HPLC) analysis confirmed the formation of a single radiolabeled species with a retention time (t_R_) of 13.9 min. This retention time closely matches the analogous rhenium complex **2**, which exhibited a t_R_ of 13.3 min, thereby validating the successful synthesis and structural integrity of the ^99m^Tc-complex. Complex **2**’ exhibits remarkable stability in Phosphate-buffered saline (PBS) (pH 7.4) at 37 °C, retaining over 95% of its original form over a 6 h incubation period. Furthermore, the complex demonstrated high stability when challenged with 2 × 10^−^^3^ M cysteine and histidine solutions at 37 °C without significant decomposition for up to 6 h. These results highlight the complex’s robust nature under physiologically relevant conditions, essential for its application in radiopharmaceuticals. The lipophilicity of the complex **2**’ was determined using the shake flask method, yielding a logD_7.4_ value of 3.2 ± 0.2. This logD_7.4_ value indicates high lipophilicity, which is favorable for efficient cellular membrane permeability. Such a lipophilicity profile is desirable for radiopharmaceutical agents, as it beneficially influences biodistribution. In the preliminary in vitro study in A431 cells, complex **2**’ showed an uptake of 12% of total activity after 30 min of incubation.

### 3.3. In Vitro Biological Studies

#### 3.3.1. Cell Growth Inhibition

**Figure 2:**
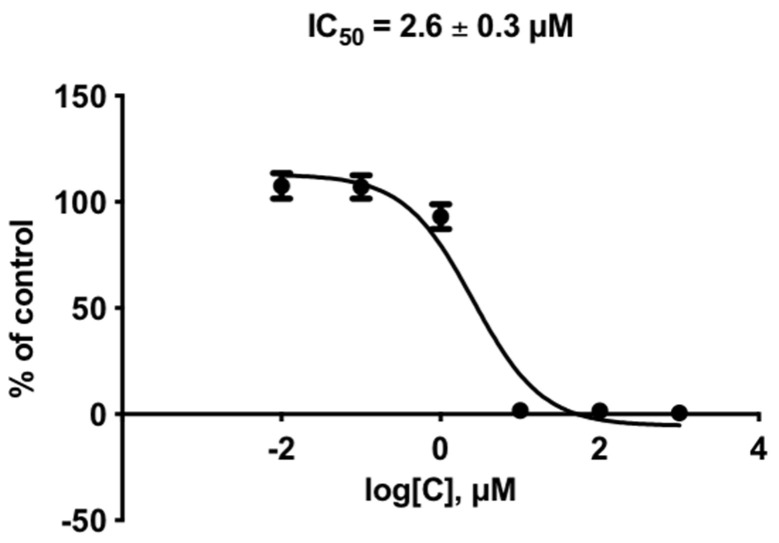
Dose−response curve from the MTT assay for complex **2** on the A431 cell line. The concentrations of the compound ranged from 0.01 to 1000 μM.

Cell growth inhibition by the novel complex was evaluated using an MTT assay on the A431 cell line overexpressing EGFR. Cytotoxicity half-maximal inhibitory concentration (IC_50_) was calculated, and, in this paragraph, the term IC_50_ refers to the cytotoxicity results. Complex **2** exhibits an IC_50_ value of 2.6 ± 0.3 μΜ. The dose–response curve is shown in Figure 2. The IC_50_ values of both complexes **1** and **2** are presented in Table 1. 

The IC_50_ value for complex **2** is 2.6 ± 0.3 μΜ, while for the already published complex **1** it is 2.0 ± 1.0 μΜ [25]. These results show that the novel compound exhibits similar cytotoxic activity with complex **1**. In addition, the IC_50_ value for the anticancer scaffold 6-amino-4-[3-bromophenyl]quinazoline is 4.8 ± 0.2 μΜ, showing that both complexes have an enhanced cytotoxic activity compared to the scaffold, which might be attributed to the metal component [22]. The cytotoxicity of the new compound is also within the range of the cytotoxic activity reported for the first generation EGFR TKIs currently used in clinical practice since, in the literature, the IC_50_ values for gefitinib, erlotinib, lapatinib, using the A431 cell line, range from 1.33 to 4.80 μΜ [34,35,36,37,38,39]. 

Additionally, in the literature, many efforts describe the formation of metal complexes combined with EGFR TKIs and tested, among others, against the A431 cell line [19]. Specifically, Jun Du et al. synthesized ruthenium(II) polypyridyl complexes combined with EGFR-inhibiting 4-anilinoquinazoline pharmacophores [40]. The MTT IC_50_ value after 48 h of treatment with the compounds ranged between 11 and 38 μΜ without EGF treatment and 13–32 μΜ with EGF treatment. Yang Zhang et al. synthesized luminescent cyclometalated platinum(II) complexes reporting at 48 h, IC_50_ values of 5–54.8 μΜ and 5.12–70.5 μΜ with and without EGF treatment, respectively [41]. Karnthaler-Benbakka et al. used prodrugs to design Co(III) and new EGFR inhibitors with dual chelating moieties, exhibiting ΙC_50_ values 9.2–> 25 μΜ after cell incubation for 72 h [39]. Our cytotoxicity results are similar to the above-reported ranges, showing the promising cytotoxic activity of the new compounds. 

#### 3.3.2. Inhibition of EGFR Phosphorylation

**Figure 3:**
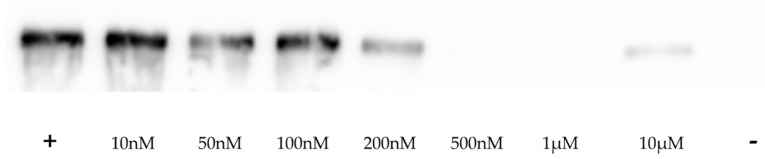
Inhibition of EGFR phosphorylation in A431 cells after treatment with complex **2** at a concentration ranging from 10 nM to 10 μΜ. Symbol + represents the positive control and refers to A431 cells with the presence of EGF but without compound treatment. Symbol − represents the negative control and refers to A431 cells without the presence of EGF and without compound treatment.

**Figure 4:**
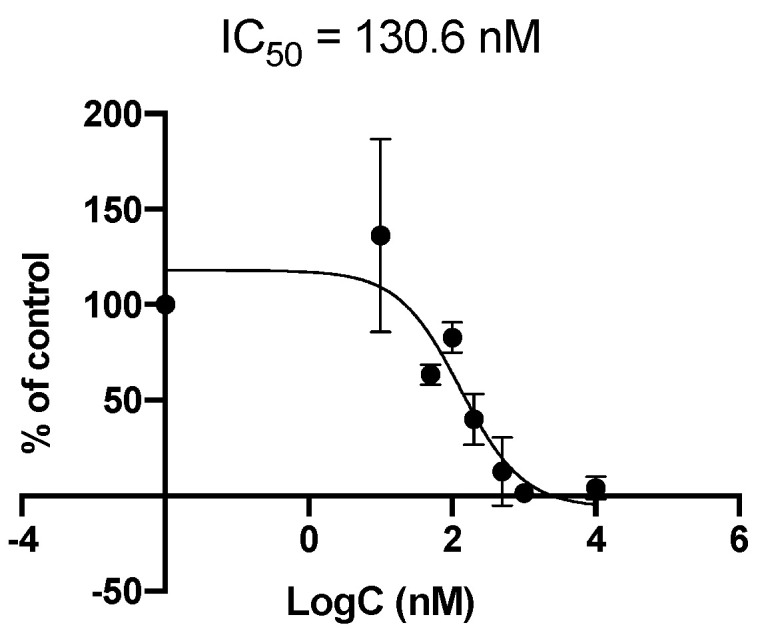
Dose−response curve for complex **2** and IC_50_ calculation of EGFR phosphorylation inhibition after Western blotting analysis (R^2^ = 0.81).

The novel complex **2** was evaluated for its potency in targeting EGFR and inhibiting phosphorylation in A431 cells. The results showed that complex **2** could cross the cell membrane and inhibit the receptor, exhibiting an EGFR phosphorylation inhibition IC_50_ value of 130.6 nM (Figure 3 and Figure 4).

Complex **1** has already been tested and was shown to inhibit EGFR with an IC_50_ value of 114 ± 23 nM [25]. The new complex maintains the ability to target EGFR, since the inhibitory activity remains similar after the replacement of Br atoms with PPh_3_.

#### 3.3.3. Cell Cycle Analysis

**Figure 5:**
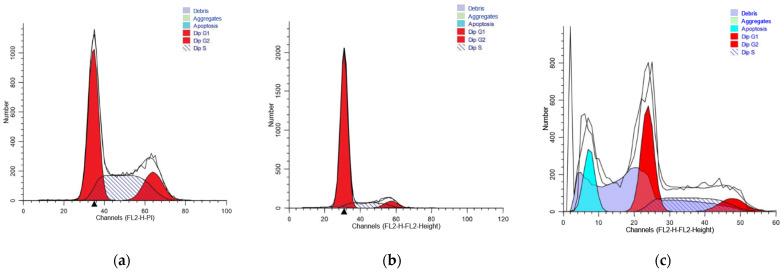
Effect of the complexes on cell cycle. (**a**) Cell cycle analysis of A431 cells without treatment (control). (**b**) Cell cycle analysis of A431 cells treated for 24 h with complex **1** at the cytotoxicity IC_50_ concentration (2 μΜ). (**c**) Cell cycle analysis of A431 cells treated for 24 h with complex **2** at the cytotoxicity IC_50_ concentration (2.6 μΜ).

To determine whether complexes **1** and **2** impact the cell cycle, A431 cells were treated with the complexes and fixed and analyzed using flow cytometry. Both complexes induce a significant G_0_/G_1_ arrest (*t*-test, *p* < 0.05). Complex **1** also shows a significant reduction in S-phase (*t*-test, *p* < 0.05). In addition, both complexes also induce a significant reduction in the G_2_/M phase. Moreover, complex **2** induces 22.98% cell death, whereas complex **1** does not exhibit the same effect. The results of the cell cycle analysis are presented in Table 2 and Figure 5. 

Clinically used drugs such as erlotinib and gefitinib induce G_0_/G_1_ arrest in A431 cells and, at the same time, decrease the proportion of cells in the S phase [42,43]. On the other hand, lapatinib induces A431 cell line arrest in the G_2_/M phase [44]. The complexes of this study were shown to induce G_0_/G_1_ arrest and to significantly increase the proportion of cells in this phase, acting similarly to gefitinib and erlotinib. 

Additionally, complex **2** induces cell death, a phenomenon likely attributed to its positive charge and the presence of the monodentate ligand PPh_3_. Positively charged lipophilic compounds with PPh_3_ are well known for their ability to target mitochondria. Triphenylphosphine plays a critical role in the structural modification of existing anticancer drugs, aiding in the design and synthesis of new mitochondrial-targeted anticancer agents [45]. Li et al. (2020) synthesized copper complexes to assess the influence of PPh_3_ on anticancer activity. Their findings revealed that the PPh_3_-containing complex disrupted mitochondrial structure, leading to the collapse of the mitochondrial membrane, ATP depletion, and Ca^2+^ leakage, ultimately inducing apoptosis [46]. Furthermore, metal complexes incorporating PPh_3_ have demonstrated the ability to mediate caspase-dependent apoptotic cell death [47,48]. Thus, our complex likely acts similarly to other positively charged triphenylphosphine compounds, effectively inducing cell death through mitochondrial disruption and subsequent apoptosis pathways.

#### 3.3.4. Influence of the Complexes on A431 Cell Line Radiosensitivity

**Figure 6:**
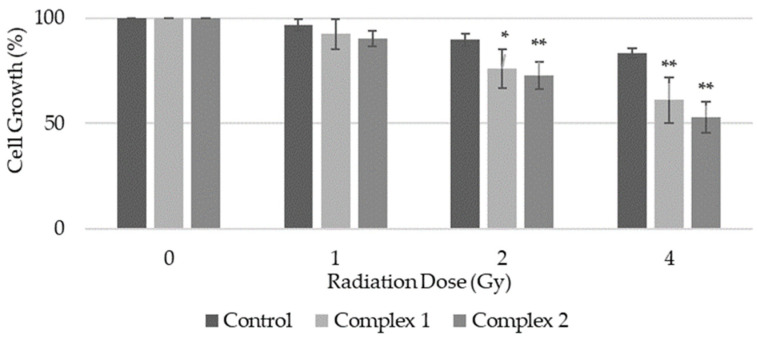
Cell growth (A431 cells) after treatment with the complexes for 24 h and irradiated at 0, 1, 2, and 4 Gy. Data are expressed as the mean ± SD from three independent experiments. ** *p* < 0.01 vs. the control of each radiation dose; * *p* < 0.05 vs. the control of each radiation dose (Student’s *t*-test).

**Figure 7:**
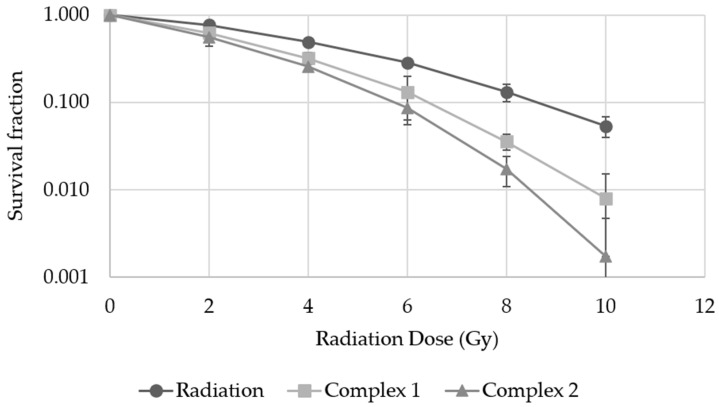
Dose–response curves after treatment with the complexes and irradiated at 0, 2, 4, 6, 8, and 10 Gy. Data are expressed as the mean ± SD from three independent experiments. The combined treatment induces significant radiosensitization at radiation doses greater than 2 Gy (Student’s *t*-test, *p* < 0.05).

The effect of the complexes on the radiosensitivity of the radioresistant A431 cells was investigated using the MTT method [29,30] and the clonogenic assay [31]. Specifically, the MTT method demonstrated that complexes **1** and **2** significantly reduced cell growth when combined with radiation at doses 2 and 4 Gy (Figure 6). Furthermore, the dose–response curves from the clonogenic assay confirmed that both complexes significantly enhance radiosensitization on the radioresistant A431 cell line, particularly at doses greater than 2 Gy (Figure 7). 

These results show that complexes combined with radiation induce a radiosensitization effect at radiation doses greater than 2 Gy (*p* < 0.05). This effect was reported at 1 μΜ concentration, even lower than the cytotoxicity IC_50_ concentration. 

In the literature, it is suggested that radiosensitization might be a consequence of the effect of the quinazolines and irradiation on the cell cycle. The complexes induce G_0_/G_1_ arrest, and, at the same time, radiation induces a strong G_2_/M arrest [49]. This leads to a decrease in the proportion of cells in the S phase, widely known as the most radioresistant phase of the cell cycle [42,50]. However, since parameters such as cell p53/p21 signaling affect the radiosensitivity profile of cancer cells, the complexes will be tested in various cell lines. Additionally, drug concentration and administration schedule also have an important role in radiosensitization [51,52,53,54]. For the reasons mentioned above, further investigation is needed to better understand the mechanisms involved in the synergistic action of the complexes with radiation. 

## 4. Conclusions

This study presents the synthesis and biological evaluation of a novel rhenium tricarbonyl complex (Complex **2**) which incorporates the EGFR TKI 6-amino-4-[3-bromophenyl]quinazoline moiety and PPh3, demonstrating notable cytotoxicity and enhanced biological activity. Complex **2**, positively charged, exhibited significant cytotoxicity against the A431 cell line with a IC50 of 2.6 ± 0.3 μM and maintained its ability to inhibit EGFR phosphorylation (IC_50_ = 130.6 nM), comparable to the previously developed neutral complex 1. Both complexes induced G_0_/G_1_ cell cycle arrest, with Complex **2** additionally promoting apoptosis. Notably, both complexes significantly enhanced radiosensitivity in A431 cells, suggesting their potential in combined modality treatments. The analogous ^99m^Tc-Complex **2**’ was successfully synthesized and characterized, exhibiting high radiochemical yield, stability, and favorable lipophilicity (LogD7.4 3.2 ± 0.2), making it suitable for radiopharmaceutical applications. A preliminary in vitro study of the 99mTc-complex in A431 cells showed a promising uptake of 12% in total activity after half an hour of incubation, indicating the potential for further in-depth in vitro and in vivo investigations. These findings underscore Complexes **2** and **2**‘s’ promise as multifaceted anticancer agents, combining effective EGFR inhibition and mitochondrial apoptosis induction, and highlight their potential for radiopharmaceutical applications in overcoming therapeutic limitations in EGFR-overexpressing cancers.

## Figures and Tables

**Table 1:** Inhibition of cell growth and cytotoxicity IC_50_ values for the two complexes on the A431 cell line overexpressing EGFR.

Compound	Cytotoxicity IC_50_ Value (μΜ) ± SD ^1^
6-amino-4-[3-bromophenyl]quinazoline	4.8 ± 0.2 [22]
Complex **1**	2.0 ± 1.0 [25]
Complex **2**	2.6 ± 0.3

^1^ Results are expressed as mean ± standard deviation (SD) from three independent experiments.

**Table 2:** Cell cycle analysis.

Complex	G_0_/G_1_	S	G_2_/M	Cell Death
Control	46.83 ± 2.32	35.83 ± 1.96	17.34 ± 1.07	0.07 ± 0.10
**1**	78.40 ± 1.57	14.39 ± 0.82	7.21 ± 0.78	0.93 ± 1.61
**2**	54.87 ± 2.21	33.73 ± 2.11	11.41 ± 0.81	22.98 ± 7.20

## Data Availability

The data presented in this study are available on request from the corresponding author.

## Supplementary Materials

The following supporting information can be downloaded at: https://www.mdpi.com/article/10.3390/pharmaceutics16091213/s1, Figure S1. IR spectra of complex **2**. Figure S2. ^1^H spectra of complex **2**. Figure S3. HPLC chromatogram of complex **2**. Figure S4. HPLC radiochromatogram of complex **2**’.
