# Supplementary material for: Synthesis and Biological Evaluation of Novel Cationic Rhenium and Technetium-99m Complexes Bearing Quinazoline Derivative for Epidermal Growth Factor Receptor Targeting
Source: Pharmaceutics. 2024 Sep 16;16(9):1213. doi: 10.3390/pharmaceutics16091213 (PMC11434983; doi:10.3390/pharmaceutics16091213)
Supplement: Supplementary file 1 [file pharmaceutics-16-01213-s001.zip › pharmaceutics-3150532-supplementary.pdf]

Supporting Information

Synthesis and biological evaluation of novel cationic Rhenium and Technetium-99m complexes bearing quinazoline derivative for EGFR targeting

Sotiria Triantopoulou[1,2], Ioanna Roupa[2], Antonio Shegani[2], Nektarios Nikolaos Pirmettis[2], Georgia Terzoudi[2], Aristeidis Chiotellis[2], Maria Tolia[3], John Damilakis[1], Ioannis Pirmettis[2*], Maria Paravatou-Petsota[2,*]

[1] Department of Medical Physics, School of Medicine, University of Crete, P.O. Box 2208, 71003 Heraklion, Crete, Greece. john.damilakis@med.uoc.gr (J.D.)

[2] Institute of Nuclear and Radiological Sciences and Technology, Energy & Safety, NCSR "Demokritos", P.O. Box 60037, 15310 Athens, Greece. iro@rrp.demokritos.gr (I.T.) ; ioanna_roupa@yahoo.gr (I.R.), ant.she@hotmail.gr (A.S.) ; nnpirmettis@pharm.uoa.gr (N.N.P.); gterzoudi@rrp.demokritos.gr (G.T.); achiotel@rrp.demokritos.gr (A.C.) ; ipirme@rrp.demokritos.gr (I.P.) ; mparavatou@rrp.demokritos.gr (M.P.P.)

[3] Department of Radiation Oncology, University Hospital of Iraklion, 71110 Iraklion, Crete, Greece. mariatolia@uoc.gr (M.T.)

[*] Correspondence: ipirme@rrp.demokritos.gr; mparavatou@rrp.demokritos.gr

Table of Contents

Figure S1. IR spectra of complex **2** 3
Figure S2. ^{1}H spectra of complex **2**........ 4
Figure S3. HPLC chromatogram of complex **2**........ 5
Figure S4. HPLC radiochromatogram of complex **2′**. 5

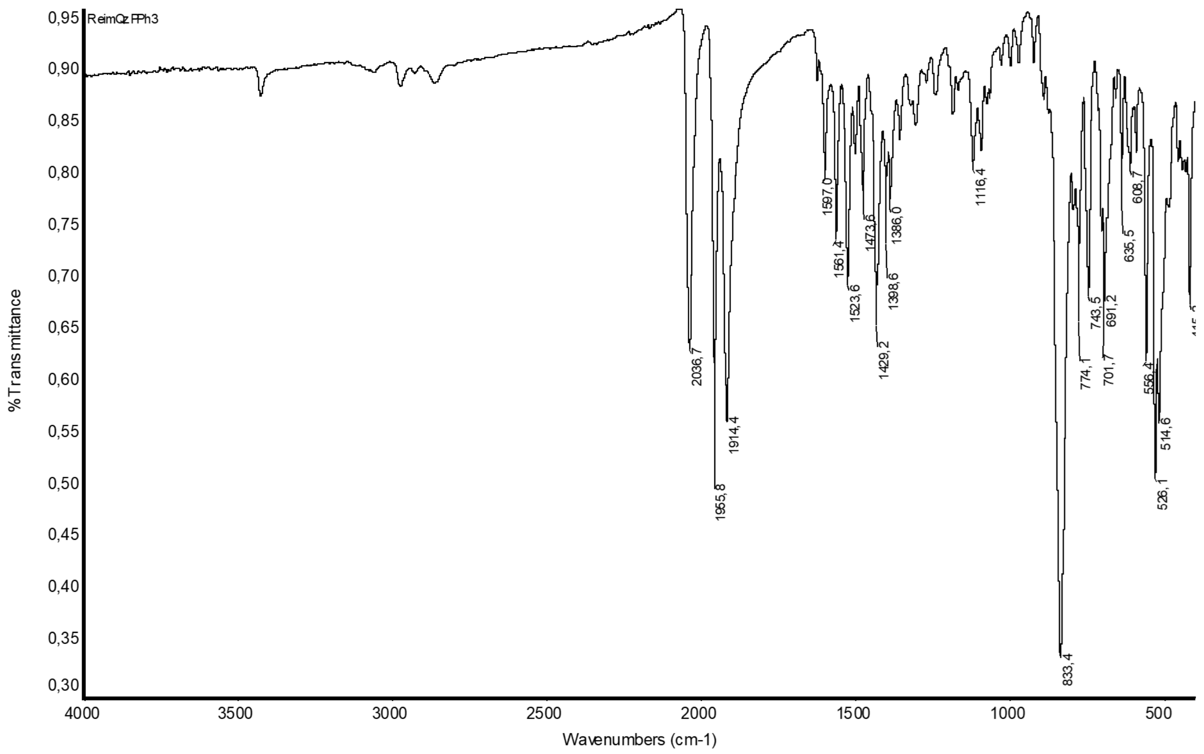

Figure S1. IR spectra of complex **2**

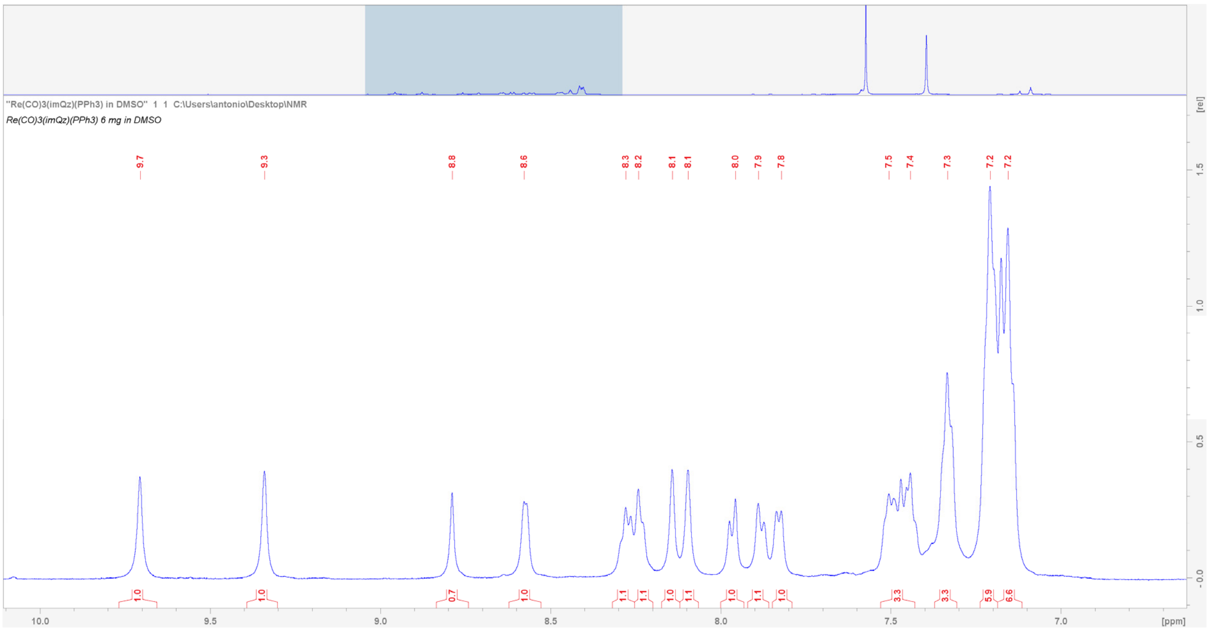

Figure S2. ^{1}H spectra of complex **2**.

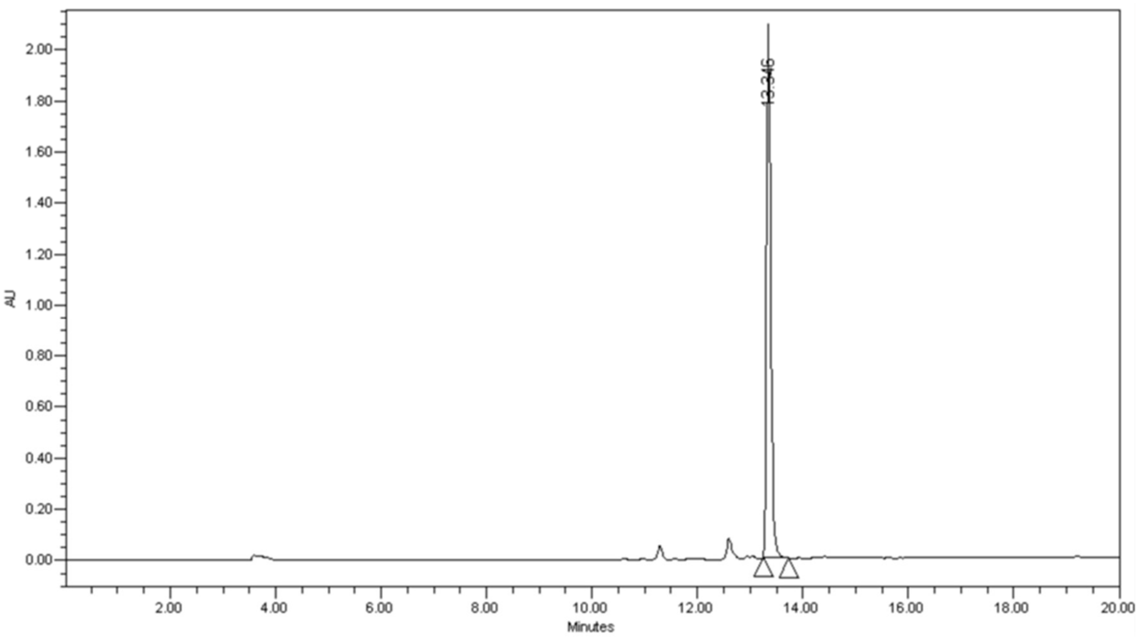

Figure S3. HPLC chromatogram of complex **2**.

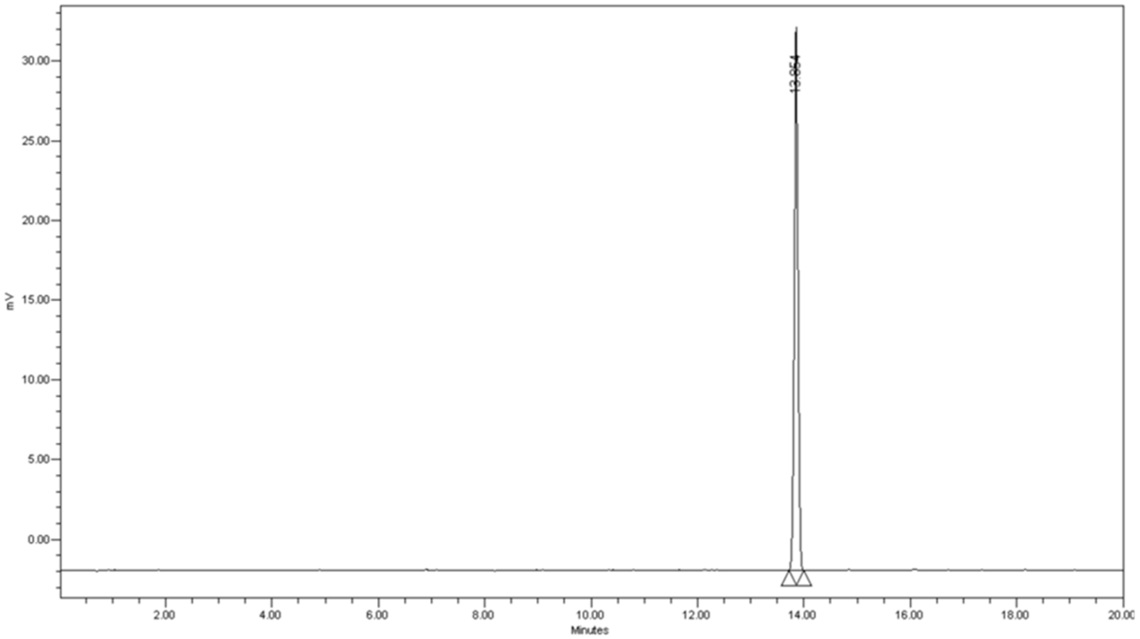

Figure S4. HPLC radiochromatogram of complex **2ʹ**.
